# Supplementary material for: Expression Profiles of Microsatellites in Fruit Tissues of Akebia trifoliata and Development of Efficient EST-SSR Markers
Source: Genes (Basel). 2022 Aug 15;13(8):1451. doi: 10.3390/genes13081451 (PMC9408125; doi:10.3390/genes13081451)
Supplement: Supplementary file 1 [file genes-13-01451-s001.zip › supplementary figures.pdf]

Supplementary Figures

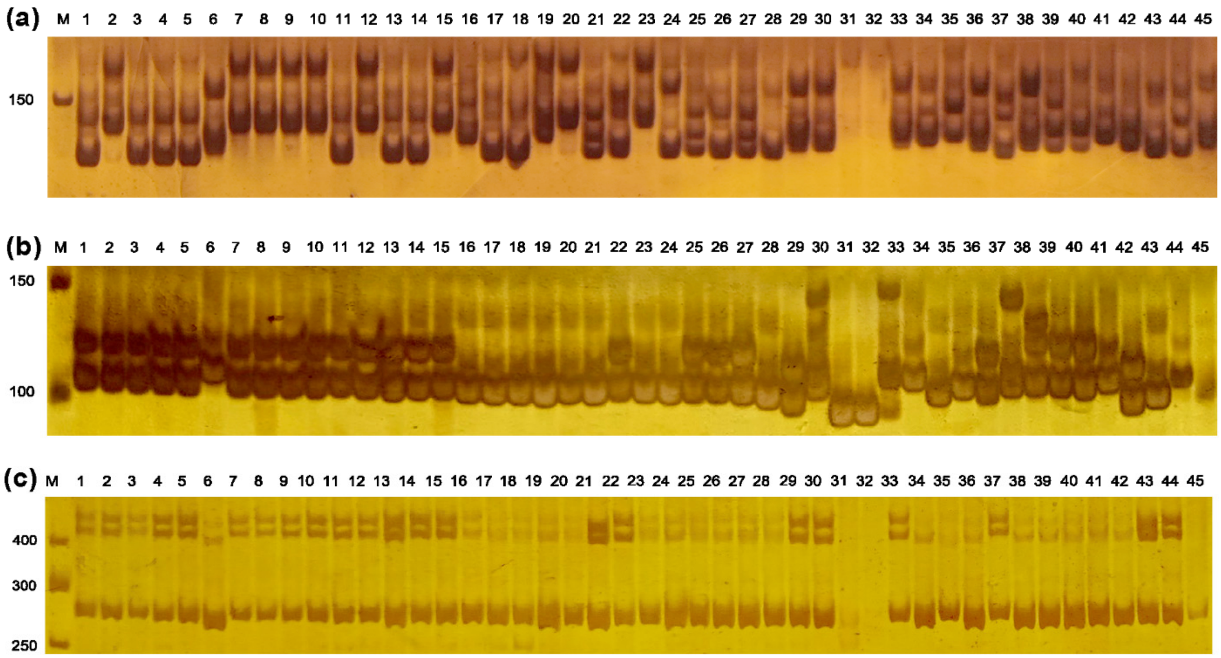

Figure S1. EST-SSR marker amplification results were separated by non-denaturing polyacrylamide gel. (a) Amplification results of markers EST-SSR-8, (b) Amplification results of markers EST-SSR-75, (c) Amplification results of markers EST-SSR-93
